# Prostatic Leiomyosarcoma: The Case for Combined Modality Therapy

**DOI:** 10.1080/13577149778498

**Published:** 1997-03

**Authors:** Salah El-Sharkawi, Keith Vaughton

**Affiliations:** 1 Department of Clinical Oncology Singleton Hospital Sketty Lane Swansea SA2 8QA UK; 2 Morriston Hospital Swansea UK

## Abstract

*Patient*. A 65-year old man who had previously undergone surgery for benign prostatic hyperplasia presented with symptoms of recurrent bladder outflow obstruction. Cystoscopy revealed the presence of recurrent tissue.

*Results*. Histology identified a leiomyosarcoma. Several years after further surgery and radiotherapy, the patient presented with secondaries in the lungs and brain, but there was no evidence of local recurrence.

*Discussion*. It is suggested that combined modality therapy (conservative surgical resection followed by adjuvant radical radiotherapy) be the initial treatment for prostatic leiomyosarcoma.


*Sarcoma* (1997) **1**, 59–60

CASE REPORT

### Prostatic leiomyosarcoma: the case for combined modality therapy

SALAH EL-SHARKAWI[1] & KEITH VAUGHTON[2]

[1]*Singleton Hospital, Swansea &* [2]*Morriston Hospital, Swansea, UK*

**Abstract**

*Patient.* A 65-year old man who had previously undergone surgery for benign prostatic hyperplasia presented with symptoms of recurrent bladder outflow obstruction. Cystoscopy revealed the presence of recurrent tissue.
*Results.* Histology identified a leiomyosarcoma. Several years after further surgery and radiotherapy, the patient presented with secondaries in the lungs and brain, but there was no evidence of local recurrence.
*Discussion.* It is suggested that combined modality therapy (conservative surgical resection followed by adjuvant radical radiotherapy) be the initial treatment for prostatic leiomyosarcoma.

**Key words**: *prostate, radiotherapy, sarcoma.*

### Introduction

Leiomyosarcoma of the prostate is a rare, highly malignant tumour, that occurs predominantly in adults. Less than 100 cases in adults have been published. We report another case and review the literature. The natural history of the tumour is characterized by rapid growth, and eventually wide dissemination to the lungs, brain, bone and liver. The prognosis remains poor regardless of therapy, with all patients dying of disseminated disease within a few years of the diagnosis. A review of the literature over the past few decades suggests that radical surgical treatment is the most commonly advocated therapeutic modality. Complete excision in most cases, however, is extremely difficult, commonly followed by recurrence, and rarely results in cure. We suggest, for patients with localized prostatic leiomyosarcoma, combined modality therapy in the form of conservative prostatic surgical resection, followed by adjuvant radical radiotherapy, as the initial treatment. Radical surgery should be reserved only for local failure and in the absence of distant metastasis.

### Patient

A 65-year-old man presented with symptoms of recurrent bladder outflow obstruction. He had undergone transurethral resection of the prostate 6 years previously for benign prostatic hyperplasia. Examination revealed a benign-feeling prostatic enlargement and cystoscopy revealed an encroachment of the prostatic cavity by recurrent tissue. Further resection was performed and histology identified a leiomyosarcoma. Further ultrasound and CT scan for staging failed to show any evidence of disease outside of the prostate. Radical prostatectomy was advised but the patient declined. Accordingly, a radical course of radiotherapy was administered to the prostate gland after a further deep resection of the prostate cavity.

The patient remained well for 3 years when he presented with dyspnoea on exertion. Chest X-ray and CT of the chest showed multiple metastases consistent with secondaries from sarcoma of the prostate. He received chemotherapy for palliation but a few months later he developed a headache and CT confirmed secondaries in the brain. Four years after the diagnosis, there was no evidence of local recurrence, but the patient died of disseminated disease in the lung and brain.

### Discussion

Prostatic sarcomas are extremely rare, constituting 0.1–0.2% of primary prostatic neoplasms. Prostatic leiomyosarcoma (PL) is the second most common prostatic sarcoma (after rhabdomyosarcoma) and accounts for most cases in adults with a median age at diagnosis of 58 years.[1] In the majority of cases, there is no clear aetiology but previous pelvic radiotherapy has been incriminated in some cases.[2]

Correspondence to: S. El-Sharkawi, Department of Clinical Oncology, Singleton Hospital, Sketty Lane, Swansea SA2 8QA, UK. Fax: + 44 1792 205776.

1357-714X/97/010059-02 ©1997 Journals Oxford Ltd

Inkeeping with our patient, the most common presenting symptoms are those of bladder neck outflow obstruction. Other symptoms include pelvic pain, haematuria and constipation.

It is impossible to make the diagnosis of PL by rectal examination as the findings are often of a smooth firm enlargement of the prostate.

In view of the rarity of PL, the diagnosis is often unsuspected at the time of presentation, and like all sarcomas, the importance of appropriate biopsy and opinion of an experienced pathologist in the diagnosis cannot be over-emphasized.[3] Ultrasound is a useful preliminary investigation, but CT scan or magnetic resonance imaging are very important, not only for evaluating the primary site but also in determining the presence or absence of metastasis and assessment of response to treatment.

Proper management of PL depends on sound knowledge of its pathology, biology and natural history, and is best achieved by close collaboration between pathologist, urologist and oncologist. The disease remains localized for a variable period of time before it spreads locally to the bladder, rectum or perineum, or to a distant location in the lungs, brain, bone and liver, hence the importance of early diagnosis and treatment.

PL is a highly malignant tumour for which curative therapy remains elusive. Because of its rarity, it is not clear from the literature whether surgery, radiotherapy, chemotherapy or a combined modality treatment offers the greatest hope. Over the last few decades, these therapeutic modalities have been attempted separately and in various combinations but none resulted in cure.[4–6] In patients with poor survival prospects like that of PL, regardless of the therapeutic modality, the aim of the treatment must be to achieve and maintain local control of the prostatic disease as long as possible with minimum morbidity. On reviewing the literature, radical surgical excision was the most frequently and performed therapeutic modality.[4,5] There is little evidence, however, that it has any curative value in the management of PL. In addition, it is associated with significant physical and psychological morbidity. Cheville *et al.* described 11 patients who underwent curative radical surgery for localized PL: five of them had gross residual tumour, five experienced local recurrence 2–41 months after surgery, and one patient had no evidence of recurrence 4.5 months after surgery.[4] Mottola *et al.* described two patients with PL who died 13 and 20 months after radical surgery due to recurrence.[7] Russo *et al.* reported a patient with PL who died of metastatic disease soon after radical surgery.[8] These cases are consistent with the experience and results of other authors.[9,10] They all indicate that PL is frequently aggressive.

PL in general has a high propensity for local recurrence and distant spread. This may be related in part to the delay in diagnosis and presentation. In the 1970s, the concept of using limited surgical excision with adjuvant radical radiotherapy (combined modality therapy) was introduced in the management of soft tissue sarcomas, and its value has been demonstrated in several sites and at different centres.[11–13] Ahlering *et al.* reported the outcome of four patients with PL treated with combined modality therapy (surgical resection and radical radiotherapy): three of them were alive with no residual disease at 60, 73 and 87 months follow-up.[14] Our patient remained recurrence free for 3 years, then died after 4 years of disseminated disease in the lungs and brain. Locally, however, there was no evidence of recurrence of his PL. Our experience with this case is consistent with the experience of others that PL is frequently aggressive but occasionally it takes an indolent course.[4] The encouraging results using conservative surgery and radical radiotherapy (combined modality therapy) require further national or international study of the role of combined modality therapy in the management of PL.

### References

1 Gilliland FD, Key CR. Male genital cancer. *Cancer* 1995; 75:295–315.

2 Nghiem HV, Sommer FG, Moretto JC. MRI of radiation-induced prostatic sarcoma. *Clin Imaging* 1995; 19:54–6.

3 Robinson MH. The management of adult soft tissue sarcomas. *Clin Oncol* 1994; 6:183–92.

4 Cheville JC, Dundare PA, Nascimento AG, *et al.* Leiomyosarcoma of the prostate. *Cancer* 1995; 76:1422–7.

5 Christoffersen J. Leiomyosarcoma of the prostate. *Acta Chir Scand Suppl* 1973; 433:75–84.

6 Schmidt JD, Welch MJ. Sarcoma of the prostate. *Cancer* 1976; 37:1908–12.

7 Mottala A, Selli C, Carini M, *et al.* Leiomyosarcoma of the prostate. *Eur Urol* 1985; 11:131–3.

8 Russo P, Brady MS, Conlan K, *et al.* Adult urological sarcoma. *J Urol* 1992; 147:1032–7.

9 Narayana AS, Loening S, Weimar GW, *et al.* Sarcoma of the bladder and prostate. *J Urol* 1978; 119:72–6.

10 Aragona F, Serretta V, Marloni A, *et al.* Leiomyosarcoma of the prostate in adults. *Ann Chir Gyn* 1985; 74:191–4.

11 Lindberg RD, Martin RG, Romsdahl MM, *et al.* Conservative surgery and post-operative radiotherapy in 300 adults with soft tissue sarcomas. *Cancer* 1981; 47:2391–7.

12 Abbatulli JS, Boulier N, de Ranieri J, *et al.* Radiotherapy as an integrated part of the treatment of soft tissue sarcomas. *Radiother Oncol* 1984; 1:115–21.

13 Suit HD, Mankin H, Schiller AL, *et al.* Results of treatment of sarcoma of soft tissue by radiation and surgery at the Massachusetts General Hospital. *Cancer Treat Symp* 1985; 3:49–57.

14 Ahlering TE, Weintraub R, Skinner DG. Management of adult sarcomas of the bladder and prostate. *J Urol* 1988; 140:1397–9.
